# Increased Intracranial Pressure in the Setting of* Enterovirus* and Other Viral Meningitides

**DOI:** 10.1155/2017/2854043

**Published:** 2017-04-12

**Authors:** Jules C. Beal

**Affiliations:** The Saul R. Korey Department of Neurology, Montefiore Medical Center and Albert Einstein College of Medicine, Bronx, NY, USA

## Abstract

Increased intracranial pressure due to viral meningitis has not been widely discussed in the literature, although associations with* Varicella* and rarely* Enterovirus* have been described. Patients with increased intracranial pressure and cerebrospinal fluid analysis suggestive of a viral process are sometimes classified as having atypical idiopathic intracranial hypertension (IIH). However, a diagnosis of IIH requires normal cerebrospinal fluid, and therefore in these cases an infection with secondary intracranial hypertension may be a more likely diagnosis. Here seven patients are presented with elevated intracranial pressure and cerebrospinal fluid suggestive of viral or aseptic meningitis. Of these, 1 had* Enterovirus* and the remainder were diagnosed with nonspecific viral meningitis. These data suggest that viral meningitis may be associated with elevated intracranial pressure more often than is commonly recognized.* Enterovirus* has previously been associated with increased intracranial pressure only in rare case reports.

## 1. Introduction

Infections involving the central nervous system have an established association with increased intracranial pressure (ICP). Bacterial meningitis and central nervous system involvement of tuberculosis, for example, are well-described to lead to elevations of ICP, as are fungal infections [1–3]. Less frequently, increased pressures have been reported in association with Lyme disease [4]. There is a general paucity of information in the literature regarding viral meningitis as a cause of increased ICP, although cases associated with* Varicella* [5] and very rarely* Enterovirus* infection [6, 7] have been described. Patients with elevated intracranial pressure and a cerebrospinal fluid (CSF) profile suggestive of an infectious process in the absence of any of these known infectious causes are sometimes characterized as having a nonspecific viral syndrome [8] or an atypical presentation of idiopathic intracranial hypertension (IIH, also known as pseudotumor cerebri). The clinical definition of IIH has been updated and revised multiple times, but all definitions have specified that in order to make a diagnosis of IIH there must be a normal CSF profile [9–11]. Therefore patients with CSF analysis suggestive of an infectious process cannot be given a diagnosis of idiopathic intracranial hypertension; more likely these patients have aseptic or viral meningitis. The purpose of this paper is to report a series of patients seen by the inpatient pediatric neurology service at a tertiary care children's hospital who were found to have increased ICP and a CSF profile suggestive of viral meningitis. The frequency with which these patients were encountered suggests that viral meningitis may be associated with elevated intracranial pressure more often than is commonly recognized.

## 2. Methods

A retrospective chart review looking for cases of viral meningitis with associated elevation of intracranial pressure was performed. Cases with a diagnosis of viral meningitis, aseptic meningitis, idiopathic intracranial hypertension, or pseudotumor cerebri were identified from an existing database of all patients seen by the inpatient pediatric neurology consultation service over the five-year period from July 1, 2009, to June 30, 2014. These charts were then reviewed to identify patients who had an elevated opening pressure during lumbar puncture, defined for screening purposes as greater than 20 centimeters of water (cm H_2_O) [10], as well as an elevated white blood cell count on CSF analysis with lymphocytic predominance. Those with evidence of bacterial meningitis (including neutrophil predominance, low CSF glucose, and/or positive bacterial cultures) were excluded. Those with evidence of viral meningitis without documentation of elevated opening pressure during lumbar puncture were excluded. Patients with abnormal findings on brain imaging that could potentially contribute to elevations in intracranial pressure were excluded. Patients presenting with confusion or altered mental status were excluded as these findings may suggest meningoencephalitis, which is more commonly known to cause elevations in intracranial pressure [12].

## 3. Results

Overall, 44 patients with a diagnosis of aseptic meningitis and 81 patients with a diagnosis of intracranial hypertension were identified. Thirteen patients met both criteria. Four of these were excluded due to the presence of confusion or altered mental status. One was excluded due to evidence of Lyme disease and one was excluded due to evidence of Rocky Mountain Spotted Fever, as these are both bacterial infections. The remaining seven patients ranged in age from 10 to 19 years old. Five of the seven were male. Patient demographics and presenting symptoms are shown in Table 1. All patients complained of headache on presentation. Three of the seven had nausea and vomiting, three had neck pain or neck stiffness, three complained of blurred vision, and one had fever on presentation. Three of the seven patients had documented papilledema, and one was documented to not have papilledema. The remaining three patients did not have a documented fundoscopic exam. All patients had unremarkable brain imaging.

Table 2 shows the opening pressures and results of CSF testing for each of the seven patients. All patients had pressures above 20 cm H_2_O, ranging from 21 cm H_2_O to greater than 55 cm H_2_O, which is the highest measurable pressure on the manometer. All patients had elevated white blood cell counts in the CSF, ranging from 13 to 270 cells/*μ*L (normal defined as less than 13 cells/*μ*L), each with 60% lymphocytes or greater. Four of the patients had clearly elevated protein levels in the CSF (normal range defined as 10–41 mg/dL).

All patients had negative bacterial cultures. Polymerase chain reaction (PCR) for* Enterovirus* was performed in six patients and was positive in one patient. Though Lyme titers were positive in one patient, this patient subsequently had a negative immunoblot for Lyme disease. Cryptococcal antigen testing was performed in two patients and was negative in both. Cultures for acid fast bacilli were negative in two patients. One patient had negative PCR for herpes simplex virus, one had negative fungal cultures, and one had negative testing for* Bartonella.*

All patients had resolution of their presenting symptoms at discharge. Five of the seven patients were subsequently lost to follow-up. The remaining two patients (patients numbers 2 and 4) who continued to be seen within the hospital system did not suffer from chronic headaches, vision loss, or other long term neurologic sequelae.

## 4. Discussion

The definition of elevated ICP in children has not been well established. Almost certainly, “normal” pressures exist on a spectrum in which the same pressure may be the normal baseline in some children but pathologically elevated in others. A measured pressure of 20 cm H_2_O, used here for screening purposes, has traditionally been considered the upper limit of normal [10]. Some authors have proposed that pressures of 25 cm H_2_O may be normal, especially in obese patients [13, 14], or that pressures between 20 and 25 cm H_2_O may be nondiagnostic [15]. It has been suggested that pressures as high as 28 cm H_2_O may be normal in pediatric patients, and revised diagnostic criteria for IIH using a pressure of 28 cm H_2_O as the upper limit of normal in children have been proposed [11]. According to these revised criteria, three of the patients described above would not be considered to have elevated ICP: patients 2, 6, and 7 all had opening pressure less than 28 cm H_2_O. Patients 6 and 7, however, both had papilledema which would suggest that their pressures were elevated relative to their normal baseline. Patient 2 was the only patient to have confirmed absence of papilledema, and therefore a diagnosis of increased ICP in this patient may be debatable.

The diagnostic criteria for Idiopathic Intracranial Hypertension have been revised multiple times. All definitions, though, specify that the diagnosis of IIH requires a normal CSF profile, normal brain parenchyma on imaging, and a normal neurologic exam with the exception of cranial nerve abnormalities referable to increased ICP, for example, a sixth cranial nerve palsy [9–11]. Patients who otherwise meet criteria for a diagnosis of IIH but are found to have a CSF leukocytosis, however, likely have a secondary intracranial hypertension due to an infectious process rather than true IIH. A similar series of patients has previously been published with presumed viral meningitis-induced intracranial hypertension [8], but in general this type of nonspecific infectious etiology often goes unreported. Other infections leading to intracranial hypertension, in contrast, are widely recognized, most notably with bacterial, fungal, and tuberculous meningitis or meningoencephalitis [1–3], as well as Lyme disease [4], and viral encephalitis [12]. Increased intracranial pressure due to viral meningitis is less commonly identified, although associations with* Varicella* and very rarely* Enterovirus* have been described [5–7]. The patients described here all had CSF profiles suggestive of an infectious process, including leukocytosis with a lymphocytic predominance and, in several cases, clearly elevated CSF protein. In the absence of symptoms of encephalitis, these findings suggest that these patients likely had viral meningitis with secondary elevations in intracranial pressure [16].

The infectious workup performed on these patients was widely variable, emphasizing the need to standardize the way in which these patients are assessed. Lyme disease titers were only tested in three of the seven patients and cultures for acid-fast bacilli were only performed in two and fungal culture in one. None of the patients were tested for* Varicella*. On the other hand, six of the seven patients were tested for* Enterovirus*.* Enterovirus* has been associated with elevated intracranial pressure, but only in very rare case reports [6, 7]. Thus patient 2 may represent a rarely reported entity, although this is also the patient in whom the diagnosis of intracranial hypertension may be debatable, as discussed above.

## 5. Conclusions

While certain infections of the central nervous system are known to lead to elevated intracranial pressure, viral meningitis as a cause of increased ICP is much less commonly described. The seven patients described here with elevated ICP and lymphocytic leukocytosis in the CSF do not meet diagnostic criteria for idiopathic intracranial hypertension but rather have secondary intracranial hypertension as a result of viral meningitis. This suggests that this entity may be more common than is typically recognized.* Varicella* and to a lesser extent* Enterovirus* have been associated with increases in ICP. Therefore, in patients with clinical signs of meningitis, elevated opening pressures on lumbar puncture and CSF lymphocytosis, testing for* Varicella* and* Enterovirus* in addition to Lyme disease and tuberculosis, may be useful. The utility of testing for other viral etiologies is unclear. Other specific viral causes of meningitis have not been clearly associated with elevated intracranial pressure in the literature. However the fact that this and other papers have reported associations between intracranial hypertension and a nonspecific, presumably viral, meningitis suggests that there are likely other causative organisms that may potentially be identified with more extensive testing.

## Figures and Tables

**Table 1:** Patient demographics and presenting symptoms.

Patient	Age (years)	Sex	Clinical presentation					Papilledema
			Headache	Nausea/vomiting	Neck pain/stiffness	Visual change	Fever	
1	19	M	**+**	**+**	−	**+**	−	Unknown
2	10	M	**+**	**+**	−	−	−	−
3	15	M	**+**	−	**+**	**+**	−	**+**
4	19	M	**+**	−	−	−	−	Unknown
5	18	F	**+**	**+**	**+**	**+**	**+**	Unknown
6	10	M	**+**	−	−	−	−	**+**
7	19	F	**+**	**−**	**+**	−	**−**	**+**

Percent of total:			100	43	43	43	14	43

**Table 2:** Results of lumbar puncture and cerebrospinal fluid testing.

Patient	Opening pressure (cm H_2_O)	CSF WBC (cells/*µ*L)	CSF lymphocytes (%)	CSF protein (mg/dL)	Positive microbial studies	Negative microbial studies^*∗*^
1	50	270	88	65	—	EV, Cr, AFB, Fung
2	22	108	71	40	*Enterovirus* PCR	—
3	>55	13	89	54	—	EV, Cr, AFB, Ly, *Bartonella*
4	43	190	60	42	—	EV
5	32	260	93	64	Lyme titer elevated; immunoblot negative	EV
6	21	30	97	30	—	EV, Ly
7	26	13	91	19	—	HSV, viral culture

^*∗*^All patients had a negative CSF bacterial culture in addition to the studies listed.

CSF = cerebrospinal fluid; WBC = white blood cells; EV = *Enterovirus* PCR; Cr = cryptococcal antigen testing; AFB = culture for acid fast bacilli; Fung = fungal culture; HSV = herpes simplex virus PCR; Ly = Lyme titers.
